# The prevalence of polypharmacy in elderly attenders to an emergency department - a problem with a need for an effective solution

**DOI:** 10.1186/1865-1380-4-22

**Published:** 2011-06-02

**Authors:** Ashis Banerjee, David Mbamalu, Sayed Ebrahimi, Arshad Ali Khan, Toong Foo Chan

**Affiliations:** 1 Emergency Department, Chase Farm Hospital, The Ridgeway, Enfield EN 8JL, Middlesex, UK; 2 Barnet & Chase Farm Hospitals Trust, Chase Farm Hospital, Enfield, Middlesex, UK

## Abstract

We studied the prevalence of polypharmacy in attenders aged 75 years and over to an emergency department (ED) in North London over a period of 1 month. We identified 467 patients in this age group. Analysis of medications being prescribed revealed at least 82 patients on medication with the potential for adverse interaction. There is a need for ED-initiated strategies to identify interactions and for pathways to allow for medication review.

## Introduction

Iatrogenic disease contributes significantly to morbidity and mortality in the elderly population [1]. The ageing population in the UK is steadily expanding, with associated increased use of prescription medications. The estimated resident population in the UK in mid-2009 was 61,792,000. Over the last 25 years the percentage of the population aged 65 and over has increased by 1.7 million from 15% in 1984 to 16% in 2009. By 2034, 23% of the population is projected to be aged 65 and over compared to 18% aged under 16. The fastest population rise has been in those aged 85 and over, from 660,000 in 1984 to 1.4 million in 2009 [2]. Emergency department (ED) attendances by those aged 75 years and over are also continuing to rise. It has been recognised that emergency presentations may be influenced by the prescription of multiple drugs. The issue needs to be revisited as part of the strategy to reduce increased pressures on hospital bed capacity in the UK, as there is a perception that iatrogenic disease may contribute to avoidable admission. These strategies should be expected to be extrapolated to other health economies.

There has been a steady rise in the use of prescription drugs in the over 60 age group in England since 1997, the overall number of prescriptions dispensed during this period rising by nearly 60% [3]. In England, 796 million prescribed items were dispensed in 2007, while 500 million items were dispensed in 1997. The steepest rise in the period was in prescriptions for statins, from less than 5 million prescriptions in 1997 to 45 million in 2007. The costs of prescribing impose a financial burden on the NHS. At least 209 of our study cohort of patients were receiving five or more prescription drugs.

We set out to look at the prevalence of over-prescribing in all patients aged 75 years and over attending our emergency department in 1 month. We suggest possible solutions, which warrant further exploration or enhancement.

## Methods

Four independent physician reviewers were used for the purposes of the study.

We used the Emergency Department computer system (FirstNet) to identify all attendees, aged 75 years or over, to the emergency department at Chase Farm Hospital, Enfield, in north London during a period of 1 month. We cross-checked the list thus obtained against that collected by the liaison health visitor for patients aged 75 years of age or over, which is funded by Enfield Primary Care Trust the commissioners of the emergency service who have a financial stake in obtaining accurate figures. The health visitor reviews all patient ED records, and makes a follow-up phone call within 1 to 3 days of attendance where indicated

We extracted the following information, using a structured form, in patients aged 75 years or over attending the ED:

• Hospital number

• Date of birth

• Sex

• Presenting symptom (s)

• Current medication list (names and dosages)

• History of fall at presentation or of recurrent falls in the past

• Disposal outcome

We defined polypharmacy as the use of five or more prescription medications, as this definition is used in the North Central London region hospitals as the working definition for identifying risk factors for falls clinic referrals. There is no currently accepted international consensus definition of polypharmacy [4,5]. We furthermore looked at the potential for drug interactions, guided by the British National Formulary appendix on interactions and by the Beers criteria [6].

## Results

The period of data collection was from 10 June to 10 July 2008. The total number of patients aged 75 years and over attending during this period was 467, with 265 females and 202 males. The age range was from 75 to 101 years, with a median age of 88 years. Of the patients, 209 (45%) were on five or more prescription drugs (see Table 1); 127 (27%) were either on no medication or had no drug history recorded in the notes. Non-recording of medication in the minors (ambulatory care area) was recognised to be an issue, usually in patients with minor injuries requiring a relatively brief intervention, who numbered 68. It is possible that this under-recording led to underestimation of the prevalence of polypharmacy.

**Table 1:** Range of numbers of prescribed medications in study population

Number of prescription drugs	Number of patients
1	26
2	29
3	35
4	41
5	40
6	36
7	34
8	27
9	22
10	19
11	11
12	6
13	4
14	6
15	1
16	1
17	2

One hundred five patients (22%) presented with a fall, which was the most frequent presenting complaint. Other presenting complaints were shortness of breath (57; 12%), chest pain (25; 5%), abdominal pain (19; 4%), confusion (17; 3.6%), being unwell (36; 7.7%) and collapse (15; 3.2%).

Of the patients on five or more documented prescription medications, 82 (39%) were on combinations that had the potential for adverse reactions. The majority related to hypotensive effects of varying combinations of ACE inhibitors, loop diuretics and calcium channel blockers. Although warfarin is used in a significant number of elderly patients, we were unable to find any drug combinations leading to potential adverse interaction with warfarin usage. This may be because of the close level of monitoring of therapeutic anticoagulation in dedicated anticoagulation clinics in the hospital, and of wider awareness of the potential for drug interactions with warfarin.

## Discussion

The demography of the UK population is changing. Currently, one fifth of the UK population is 60 years or older. Increasing age is associated with changes in pharmacokinetics and pharmacodynamics, affecting the absorption, distribution, metabolism and excretion of drugs [7]. The altered physiology of old age is related to reduced total body water, reduced lean body mass and body fat, reduced serum albumin and altered protein binding, reduced liver phase one metabolism, reduced renal plasma flow, reduced glomerular filtration rate and renal clearance.

A meta-analysis identified that around 20% of people over 70 take five or more drugs [8]. These drugs are usually prescribed for co-morbidities resulting from musculoskeletal, cardiovascular, gastrointestinal, neurological and urological disorders. Polypharmacy is associated with increases in drug-drug interactions, adverse drug reactions, disease-drug interactions and food-drug interactions. There is also an increase in prevalence of falls [9], hospital admission rates, lengths of hospital stay, readmission rates and mortality rate. Associated problems include medication administration errors and poor compliance.

Adverse drug reactions can either singly or in combination precipitate an emergency department visit. They include confusion, electrolyte disorders, gait disorder and falls, postural hypotension and falls, gastrointestinal bleeding, incontinence, hypothermia and constipation [7].

In our study, 82 patients prescribed five or more prescription medications had the potential for adverse drug reactions. However, our study design does not allow for correlation of polypharmacy with the presenting complaint, as data were collected retrospectively and also because for any given presentation there may be the coexistence of multiple factors contributing to the presentation. The study highlights the emergency department as a place where potential drug interactions can be identified in high-risk elderly attenders.

The emergency department provides an environment in which polypharmacy can be identified, including its role in precipitating hospital attendance, leading to corrective action being initiated, particularly in patients being sent home [10]. In the current climate of bed shortages, emergency department gridlock and admission avoidance schemes, the presence of a, ED pharmacist would be of potential benefit to the process of identification of drug interactions [11,12]. Furthermore, rational prescribing for the elderly should be guided by consensus criteria, such as those developed in the US by Delphic methodology [13]. These essentially involve listing potentially inappropriate medications, where the risks of administration may outweigh the benefits of administration.

In our own population, we suggest more effective surveillance of prescription medication in elderly attenders to the ED, and the need for mechanisms to detect the need for, and achieve, corrective action where indicated.

**Potential strategies **Box 1

• Medication review for all ED attenders, aided by dedicated ED pharmacist sessions

• IT-based solutions to highlight potential drug interactions: electronic prescribing support systems

• Effective prescription monitoring in the community

• Targeted feedback to general practitioners to consider reducing prescription medication via care of elderly liaison health visitor

• Effective case management of chronic disease in the community

• Awareness of risk-inducing prescriptions (box 2)

**Examples of drugs that pose a particular risk for older people **Box 2

• Long term non-steroidal anti-inflammatory drugs

• Long-acting benzodiazepines, e.g. diazepam

• Anti-cholinergic drugs

• Tricyclic antidepressants

• Doxazosin

• Metoclopramide

## Competing interests

The authors declare that they have no competing interests.

## Authors' contributions

AB conceived the idea for the study; AB and DM designed the study and the data collection proforma; TFC assisted with collation of the data; AB, DM, AAK and SE actively collected the data from the departmental records. All the data have been verified by DM and AB.

## Authors' information

Ashis Banerjee has been a consultant in emergency medicine in London for the preceding 16 years, and is lead clinician at Chase Farm Hospital and honorary senior lecturer at University College London Medical School.

David Mbamalu is a consultant in emergency medicine at Chase Farm Hospital, Enfield.

Sayed Ebrahimi and Arshad Ali Khan are specialty doctors in emergency medicine at Chase Farm Hospital, Enfield.

T.F. Chan is chief pharmacist at Chase Farm Hospital, Enfield.
